# Idiopathic Hypereosinophilic Syndrome: A Case Report

**DOI:** 10.7759/cureus.39964

**Published:** 2023-06-05

**Authors:** Margarida Agudo, Francisca Santos, Ana Teixeira Reis, Pedro Moura, Susana Marques

**Affiliations:** 1 Internal Medicine, Centro Hospitalar de Setúbal, Setúbal, PRT; 2 Medicine, Centro Hospitalar Do Médio Ave, Vila Nova de Famalicao, PRT

**Keywords:** azathioprine, corticosteroid therapy, myo-pericarditis, hypereosinophilia, idiopathic hypereosinophilic syndrome

## Abstract

Idiopathic hypereosinophilic syndrome is a rare condition characterized by persistent severe eosinophilia and organ damage without any apparent cause.

A 20-year-old male patient with no significant medical history was admitted to the Emergency Department with retrosternal chest pain, fatigue and asthenia. EKG showed ST elevation I, II, III, aVF, V4-V6 and blood tests showed elevated troponin levels. An echocardiogram was performed revealing severe global left ventricular systolic dysfunction. Further evaluations included cardiac magnetic resonance imaging and endomyocardial biopsy, confirming the diagnosis of eosinophilic myocarditis. The patient was started on systemic corticosteroid therapy, resulting in clinical improvement. The patient was discharged after 12 days of hospitalization, following a recovery of biventricular function and he was told to continue oral corticosteroid therapy at home. Further investigation ruled out other causes of hypereosinophilic syndromes, therefore the diagnosis of idiopathic hypereosinophilic syndrome was assumed. Despite the attempt to reduce corticosteroid therapy, the eosinophil count became elevated again, so the dosage was increased and associated with azathioprine with subsequent favorable analytical evolution.

This case highlights the challenges in diagnosing and managing idiopathic hypereosinophilic syndrome and emphasizes the importance of prompt treatment initiation to prevent complications.

## Introduction

Eosinophils are a type of granulocytes that belong to the myeloid cell lineage. They possess cytoplasmic granules containing cytotoxic proteins, which, upon release, can directly cause tissue damage. While the majority of eosinophils are found in mucosal tissues exposed to the environment, they can also circulate in the peripheral blood. Eosinophilia is defined as an elevated eosinophil count exceeding 500 microliters (μL). Eosinophilia can be asymptomatic or give rise to a wide range of pathological conditions [1].

In 1968, Hardy and Anderson introduced the term "hypereosinophilic syndrome" to describe a group of conditions characterized by persistently severe eosinophilia and organ damage [2,3]. This syndrome exhibits heterogeneity in its etiology, which can include allergic, parasitic, and non-parasitic infections, autoimmune disorders, myeloproliferative diseases, and neoplasms. In rare cases, it can be considered idiopathic. In this way, in 1975, empirical diagnostic criteria for idiopathic hypereosinophilic syndrome were established, which include: 1) a peripheral blood eosinophil count exceeding 1500 cubic millimeters (mm3) for more than six months, 2) organ dysfunction or damage, and 3) the absence of identifiable causes for eosinophilia and eosinophilic blasts in the peripheral blood [3-9]. Given that this syndrome is a diagnosis of exclusion, all three criteria must be met to confirm idiopathic hypereosinophilic syndrome.

In terms of epidemiology, this syndrome predominantly affects males and has bimodal distribution between 20 and 50 years (although it can occur in children) [1,9]. It can involve any organ, including the skin, lungs, gastrointestinal system, central and/or peripheral nervous system [8]. Cardiac involvement is observed in 50-75% of cases and can manifest as restrictive cardiomyopathy, valvular injury, and/or heart failure. The inherent hypercoagulable state in this disease increases the risk of thromboembolic complications. The main objective of therapy is to control eosinophil count in order to prevent progression of organ damage. Numerous studies have been conducted on the treatment of hypereosinophilic syndromes, including idiopathic hypereosinophilic syndrome. Systemic corticosteroid therapy is the first-line treatment for most patients. A favorable response to prednisolone (1 mg/kg/day) initially is considered a positive prognostic indicator of a favorable course of the disease. In cases refractory to corticosteroid therapy or with more severe multiorgan involvement, cytostatics (hydroxyurea) and/or immunomodulators (IFN-α, cyclosporine) may be employed. Recently, treatment with biological therapies such as mepolizumab (a monoclonal antibody against IL-5) has been approved [1,4]. Other therapies, including JAK 2 and FGFR1 inhibitors, as well as benralizumab, are being investigated for their effectiveness [1]. In cases involving cardiac and/or thromboembolic complications, anticoagulation and cardiac surgery may be warranted [9]. The prognosis of idiopathic hypereosinophilic syndrome varies depending on its presentation, with cardiac involvement associated with a poorer prognosis and unfavorable outcomes if treatment is not initiated early.

## Case presentation

A 20-year-old Caucasian male patient presented to the emergency room with a past medical history of allergic rhinitis but no other significant medical conditions or drug allergies. The patient has his national immunization plan updated and has taken four doses of the covid-19 vaccine. He had no relevant family history, and he didn't regularly use drugs, tobacco, alcohol, or prescription medications. There was no contact with animals, and the patient had not recently traveled abroad or stayed in a rural area.

The patient reported experiencing retrosternal chest pain for the past three days, described as oppressive, intense and pleuritic. The patient didn't experience any alleviating or relieving factors for the pain. He also complained of easy fatigue with minimal exertion and overall weakness. However, he denied fever, dyspnea, cough, gastrointestinal symptoms, or any other systemic symptoms.

Upon physical examination, the patient was hemodynamically stable but had mild tachycardia (heart rate of 110 bpm). He had no fever, and cardiac auscultation revealed a regular rhythm without murmurs. An electrocardiogram (EKG) showed ST segment elevation in leads I, II, III, aVF, and V4-6. Laboratory tests indicated elevated levels of troponin I (11635 picograms per milliliter (pg/mL) - reference range: 13.8-17.5 pg/mL), but no other significant abnormalities were noted in the complete blood count, renal function, electrolytes, liver enzymes, or inflammatory markers. Testing for the SARS-CoV-2 virus came back negative. Subsequent echocardiography revealed severe systolic dysfunction of the left ventricle, with a left ventricular ejection fraction (LVEF) of 25%, showing diffuse and severe hypokinesis, particularly in the anterior and inferior walls. Additionally, right ventricular dysfunction and moderate pericardial effusion were observed. Coronary angiography did not show any evidence of coronary artery disease, leading to a provisional diagnosis of myopericarditis. The patient was transferred to a cardiac intermediate care unit for further management and investigation into the underlying cause.

**Figure 1:**
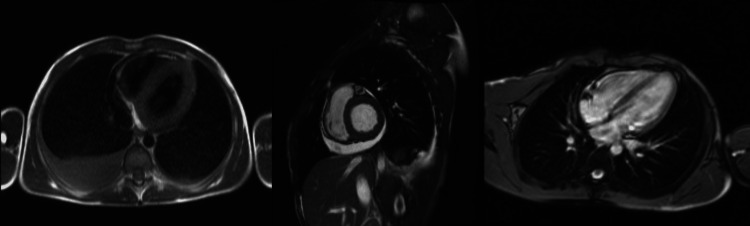
Cardiac magnetic resonance imaging with acute myocarditis

Within 24 hours of admission, the patient developed signs of low cardiac output and hyperlactatemia (with a maximum of 3 mmol), requiring the initiation of norepinephrine and dobutamine support until achieving clinical stability. Repeat echocardiography during this period showed similar findings to the previous examination. Cardiac magnetic resonance imaging (MRI) confirmed the diagnosis of acute myopericarditis, associated with moderate pericardial effusion and bilateral pleural effusion, with greater involvement on the right side. Figure 1 in the article corresponds to cardiac MRI sections illustrating the presence of myocarditis.

Further laboratory evaluation revealed a decreasing trend in troponin I levels (506 pg/mL), elevated NT-proBNP (5086 pg/mL), and a gradual increase in eosinophil count on the complete blood count (1550 μL to 2900 μL - reference range: < 1000 uL). All other laboratory tests were within normal limits. To investigate the underlying cause, an endomyocardial biopsy was performed, which showed areas of necrosis and interstitial and perivascular inflammatory infiltrates with numerous eosinophils, suggestive of eosinophilic myocarditis.

Given the persistent elevation of peripheral eosinophilia, systemic corticosteroid therapy was initiated at a dose of 1 mg/kg/day, resulting in significant clinical improvement. As a result, the need for vasopressor and inotropic support decreased and eventually ceased, allowing the patient to be started on beta-blocker therapy, angiotensin receptor-neprilysin inhibitor (ARNI), and spironolactone, all of which were well-tolerated.

Simultaneously, additional diagnostic tests were conducted to investigate the etiology. The findings of the serum study (Table 1), infectious disease study (Table 2), autoimmune study (Table 3), and imaging study (Table 4) are summarized, providing a comprehensive assessment of the patient's condition.

**Table 1:** Serum study performed during hospitalization

Serum Study	
Erythrocyte Sedimentation Rate	19 mm/h (reference range: 1-10 mm/h)
Peripheral Blood Smear	Negative
Immunoglobulins (IgA, IgM, and IgG)	Normal (IgA reference range: 70-400 mg/dL; IgM reference range: 40-230 mg/dL; IgG reference range: 700-1600 mg/dL)
Protein Electrophoresis	Slight elevation of alpha 1 and 2 (alfa 1 reference range: 2.9-4.9 g/dL; alfa 2 reference range: 7.1-11.8 g/dL)
Beta-2 Microglobulin	Negative
Free Kappa and Lambda Light Chains	Free Kappa Light Chain 1.46 mg/dL (reference range: 0.33-1.94 mg/dL); Free Lambda Light Chain 1.36 mg/dL (reference range: 0.57-2.63 mg/dL)
Total IgE	2356 kU/L (reference range: < 120 kU/l)
Bone Marrow Examination	16% eosinophils and 0.5% blasts (reference range: eosinophils <1%; blasts <1%)

**Table 2:** Infectiology study performed during hospitalization

Infectiology		
Anti-Streptolysin O Antibodies		Negative
Toxoplasmosis IgM and IgG Antibodies		Negative
Cytomegalovirus (CMV) IgM and IgG Antibodies		Negative
Epstein-Barr Virus (EBV) IgM and IgG Antibodies		Negative
Herpes Zoster Antibodies		Negative
Anti-Mycoplasma Antibodies		Negative
HIV Antigen and Antibodies		Negative
Hepatitis B Surface Antigen (HBsAg) and Anti-Hepatitis B Core Antibodies (anti-HBc)		Negative
Hepatitis C Virus (HCV) Antibodies		Negative
Anti-Hepatitis A Virus (HAV) IgM and IgG Antibodies		Negative
Anti-Treponema Antibodies and TPHA		Negative
Giardia Lamblia (Stool)		Negative
Cryptococcus Parvum (Stool)		Negative
Entamoeba Histolytica (Stool)		Negative
Parasitological Study (Stool)		Negative

**Table 3:** Autoimmunity study performed during hospitalization

Autoimmunity		
Anti-Transglutaminase IgA Antibodies		Negative
Anti-Gliadin IgM and IgG Antibodies		Negative
Rheumatoid Factor		Negative
Antinuclear Antibodies (ANA)		Negative
Anti-dsDNA Antibodies		Negative
cANCA and pANCA		Negative
Anti-Myeloperoxidase (MPO) Antibodies		Negative
Anti-PR3 Antibodies		Negative
Anti-Glomerular Basement Membrane Antibodies		Negative
Complement (C3 and C4)		Normal

**Table 4:** Imaging study performed during hospitalization

Imaging Study	
Chest CT (Pulmonary)	Normal
Paranasal Sinus CT	Moderate mucosal hypertrophy of the middle and lower turbinates in the context of rhinitis
Colonoscopy	Normal

On the 11th day of hospitalization, the patient underwent a follow-up echocardiogram, which revealed significant improvement in biventricular function with a left ventricular ejection fraction (LVEF) of 63% and mild pericardial effusion. Analytically, there was also an improvement in eosinophil count (900/μL). Based on these positive developments, the patient was discharged and referred to the Hematology and Cardiology clinics for further management. The prescribed medications during hospitalization, including beta-blocker, angiotensin receptor/neprilysin inhibitor (ARNI), spironolactone, and oral corticosteroid therapy with prednisolone 40 mg/day in a tapering regimen, were continued.

After one month of discharge, the patient began quarterly reassessments in the Cardiology clinic. During these follow-up visits, the patient remained hemodynamically stable, with no significant changes observed on physical examination and no need for adjustments to cardiovascular therapy. Six months after discharge, a cardiac magnetic resonance imaging was performed, which demonstrated the recovery of biventricular function and the absence of myocardial edema or active inflammation. Nine months after discharge, a follow-up echocardiogram confirmed sustained improvement, showing recovery of cardiac function with no residual effects (LVEF of 60%). Laboratory analysis revealed negative troponin I levels and NT-proBNP levels (27 pg/mL), indicating a favorable prognosis.

From a hematological perspective, the patient continued to be followed by the Hematology specialty with quarterly appointments. During these evaluations, periodic laboratory analyses revealed a worsening peripheral eosinophil count (1500/μL) while on a maintenance dose of prednisolone 5 mg after three months of discharge. Consequently, the decision was made to increase the corticosteroid therapy to prednisolone 10 mg/day, resulting in a sustained worsening of peripheral eosinophilia (1600/μL → 1700/μL → 1885/μL). Recognizing this gradual increase in eosinophil count and following discussions within the medical team, it was decided to resume the previous corticosteroid dosage upon discharge (prednisolone 40 mg), leading to a subsequent improvement in eosinophilia (1000/μL → 800/μL).

The additional hematological investigation is summarized in Table 5.

**Table 5:** Additional Hematological Investigation performed at Hematological clinic

Additional Hematological Investigation	
Bone Marrow Examination	9% eosinophils
Bone Biopsy	No abnormalities
Karyotype Analysis	No abnormalities
PDGFRalpha and PDGFRbeta Genes	FISH (Fluorescence In Situ Hybridization) test negative
FLT3 Gene Mutations	Negative
V617F Mutation in JAK2 Gene	Negative
BCR-ABL Fusion Transcripts	Negative
T-Cell Clonality Studies	Negative
Immunophenotyping	No abnormalities

Based on the investigations conducted both during hospitalization and outpatient care, all potential causes of hypereosinophilic syndrome have been ruled out. This suggests the presence of idiopathic eosinophilic syndrome. Given the need for high-dose corticosteroid therapy (prednisolone 40 mg), it was decided to introduce azathioprine at a dosage of 1 mg/kg/day after one year of hospital discharge.

After three months of this therapeutic change, the patient continued to experience a decrease in eosinophil count, which allowed for a gradual tapering of corticosteroid therapy. This tapering is necessary to avoid the potential iatrogenic effects associated with long-term high-dose corticosteroid use.

**Figure 2:**
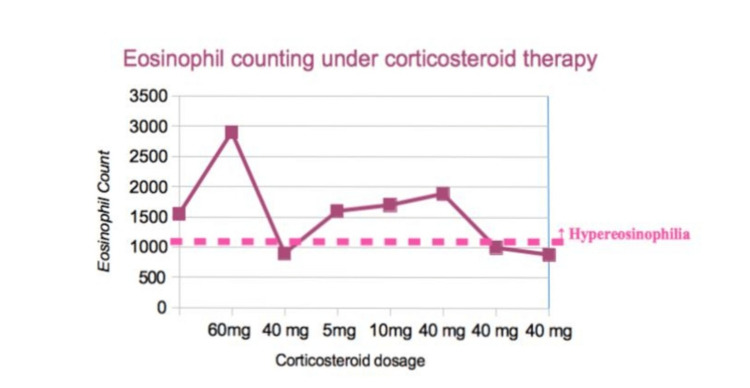
Eosinophil counting under corticosteroid therapy

Figure 2 illustrates the relationship between eosinophil count and corticosteroid dosage with time [5].

The patient maintained regular follow-up appointments with both the cardiology and hematology departments to ensure ongoing clinical surveillance and laboratory monitoring.

## Discussion

Based on the patient's age and the absence of significant personal history, the initial presentation of eosinophilic syndrome may seem unlikely. Furthermore, the lack of relevant medical history and clinical signs related to eosinophilia highlights the fact that systemic hypereosinophilic syndrome is a diagnosis that requires ruling out other potential causes. As mentioned earlier, there are various possible etiologies for eosinophilia, as outlined in Table 6 [4,9].

**Table 6:** Causes of eosinophilia

Etiology	Example
Allergic and/or atopic	Allergic rhinitis; Atopic dermatitis; Urticaria Asthma; Drug reactions (e.g., to antibiotics or nonsteroidal anti-inflammatory drugs); Episodic angioedema with eosinophilia
Infectious Non-Parasitic	Brucellosis Aspergillosis; Infectious lymphocytosis; Infectious mononucleosis; Mycobacterial disease; Scarlet fever
Parasitic Infectious	Ascariasis; Clonorchiasis; Cystocerciasis; Echinococcosis; Fascioliasis; Filariasis; Hookworm infection Paragonimiasis; Schistosomiasis; Strongyloidiasis; Toxocariasis; Trichinosis; Trichuriasis
Connective tissue, vasculitic, or granulomatous disorders	Allergic bronchopulmonary aspergillosis; Eosinophilic granulomatosis with polyangiitis; Loffler's Syndrome Eosinophilic fasciitis Idiopathic; Eosinophilic synovitis; Inflammatory bowel disease; Polyarteritis nodosa; Dressler Syndrome; Rheumatoid arthritis; Sarcoidosis Sjogren's Syndrome; Systemic lupus erythematosus
Myeloproliferative Disorders	Acute or chronic eosinophilic leukemia; Acute Lymphoblastic Leukemia; Chronic myeloid leukemia; Hypereosinophilic syndrome
Neoplasic	T cell lymphoma; Carcinomas and sarcomas of the lung, pancreas, colon, cervix, or ovary Hodgkin's Lymphoma; Non-Hodgkin's lymphomas
Primary immunodeficiencies	Hyper-IgE Syndrome; Omen syndrome

In general, parasitic infections are the most common cause of eosinophilia globally, while allergic conditions predominate in developed countries. The diagnosis of idiopathic hypereosinophilic syndrome requires a thorough investigation to rule out other potential causes of eosinophilia. In this case, the detection of eosinophilia in peripheral blood was an incidental finding that was later confirmed by endocardial biopsy.

After excluding coronary artery disease and establishing a diagnosis of myocarditis, infectious causes were initially investigated, even in the absence of fever. Despite a comprehensive targeted investigation yielding negative results and a persistent increase in eosinophilia, the decision was made to initiate systemic corticosteroid therapy early, without the risk of exacerbating any potential underlying infectious pathology.

Organ damage in eosinophilic syndromes is a direct consequence of eosinophil accumulation in tissues. In cases of cardiac involvement, eosinophils are initially deposited in myocardial cells and, once activated locally, continue to produce and secrete cytotoxic proteins that contribute to cardiac and capillary endothelial injury. However, the severity of complications does not appear to be solely related to peripheral blood eosinophilia but rather to the number of eosinophils undergoing degranulation in tissues. Complications are common among different conditions associated with eosinophilia and seem to be independent of the initial cause of eosinophilic infiltration. In cases of cardiac involvement, approximately 58% of patients are at risk of fatal complications. Three stages of injury are typically distinguished: an early necrotic stage (rarely symptomatic), a thrombotic stage with the development of intracavitary thrombus on the damaged endocardium, and a final stage of endomyocardial fibrosis, restrictive cardiomyopathy, valve damage, and congestive heart failure [3,7,9]. In this patient, serial echocardiograms and cardiac MRI revealed myocardial damage and impaired function, manifested by severely depressed ejection fraction. Therefore, it was crucial to initiate targeted corticosteroid treatment to rapidly reverse the established condition, followed by further complementary studies.

Although allergic conditions are the leading cause of hypereosinophilia in developed countries and the patient has a history of allergic rhinitis, it does not provide a definitive explanation for the severity of this presentation. Similarly, considering the patient's age, autoimmune conditions would be the third most likely hypothesis, but investigation of relevant markers and antibodies yielded negative results. While the patient did not exhibit constitutional symptoms, hypereosinophilia is often associated with neoplastic disorders. However, extensive imaging studies conducted during hospitalization did not reveal any evidence of neoplasms. In the hematology consultation, this possibility was further explored, and myeloproliferative and/or myelodysplastic processes were ruled out. In hypereosinophilic syndromes, the overproduction of eosinophils can be attributed to two distinct mechanisms: 1) clonal eosinophil proliferation due to alterations in the hematopoietic lineage and/or receptors involved in eosinophil production; 2) overproduction of eosinophilopoietic cytokines (such as IL-5). These alterations are commonly observed in myeloproliferative and T-cell lymphoproliferative variants, often accompanied by specific genetic abnormalities (PDGFRA, PDGFRB, FGFR1, PCM1-JAK2) [1]. Therefore, a bone marrow examination, bone biopsy, and extensive genetic studies were performed, all yielding negative results [1]. Consequently, after excluding all other potential causes, idiopathic hypereosinophilic syndrome was considered based on the fulfillment of the necessary criteria: 1) peripheral blood eosinophil count above 1500/mm3; 2) organ damage and dysfunction; 3) absence of an identifiable cause for eosinophilia and absence of eosinophilic blasts in peripheral blood.

Idiopathic hypereosinophilic syndrome is a diagnosis of exclusion, leading to clinical heterogeneity and variable prognosis [5,10]. The disease can present in a wide range of ways, from asymptomatic cases that do not require treatment and have long-term survival to rapidly fatal cases due to complications, particularly cardiac and hematological complications. In this case, the early initiation of systemic corticosteroid therapy resulted in a decrease in peripheral blood eosinophil levels and rapid clinical improvement, indicating a favorable prognosis. However, during follow-up, it was observed that reducing the corticosteroid dosage led to an increase in peripheral blood eosinophil count, although without clinical deterioration. Despite corticosteroid therapy being the first-line treatment and the patient showing a good response, a high dose of corticosteroids (prednisolone 40 mg/day) was required, prompting the decision to introduce an immunomodulator (azathioprine) to control the disease and mitigate the iatrogenic effects of prolonged corticosteroid use in a young patient.

It is worth noting that the prognosis of idiopathic hypereosinophilic syndrome has improved over time, primarily due to therapeutic advancements such as monoclonal antibodies. In selected cases, cardiac transplantation may be considered.

In the future, despite the patient remaining asymptomatic and experiencing significant improvement, ongoing monitoring is necessary, including regular complementary examinations, to detect any new organ damage or potential complications. It is important to maintain a high index of suspicion for any changes that may warrant reconsideration of the diagnosis. There have been reports in the literature of cases initially assumed to be idiopathic hypereosinophilic syndrome but later identified as myelodysplastic or proliferative disorders, emphasizing the need for reassessment of treatment.

## Conclusions

Indeed, this case serves as a reminder that clinical signs and medical history may not always provide clear guidance for diagnosis, emphasizing the need for a comprehensive investigation of unlikely causes. It is important to have a thorough understanding of the various etiologies of hypereosinophilia and to pursue their investigation to reach an accurate diagnosis. Cardiac involvement in hypereosinophilic syndrome can manifest in diverse ways, further complicating the diagnosis. The clinical course of eosinophilic myocarditis can vary significantly, occasionally presenting with severe symptoms. Therefore, early initiation of treatment is crucial when there is suspicion of the condition, aiming to improve the prognosis.

Regular and vigilant monitoring is essential, maintaining a high level of suspicion for any new organ damage or potential complications. However, further research is needed to deepen our understanding of the molecular basis and pathophysiology of hypereosinophilic syndrome, as well as to identify distinct nosological entities, in order to enhance therapeutic options and improve patient prognosis.
